# Fetal Neuroprotection by Magnesium Sulfate: From Translational Research to Clinical Application

**DOI:** 10.3389/fneur.2018.00247

**Published:** 2018-04-16

**Authors:** Clément Chollat, Loïc Sentilhes, Stéphane Marret

**Affiliations:** ^1^INSERM U1245, Team 4 Neovasc, School of Medicine of Rouen, Institute of Innovation and Biomedical Research, Normandie University, Rouen, France; ^2^Department of Neonatal Intensive Care, Port-Royal University Hospital, APHP, Paris, France; ^3^Department of Obstetrics and Gynecology, Bordeaux University Hospital, Bordeaux, France; ^4^Department of Neonatal Pediatrics and Intensive Care – Neuropediatrics, Rouen University Hospital, Rouen, France

**Keywords:** magnesium sulfate, neuroprotection, preterm birth, cerebral palsy, animal studies, randomized controlled trials

## Abstract

Despite improvements in perinatal care, preterm birth still occurs regularly and the associated brain injury and adverse neurological outcomes remain a persistent challenge. Antenatal magnesium sulfate administration is an intervention with demonstrated neuroprotective effects for preterm births before 32 weeks of gestation (WG). Owing to its biological properties, including its action as an *N*-methyl-d-aspartate receptor blocker and its anti-inflammatory effects, magnesium is a good candidate for neuroprotection. In hypoxia models, including hypoxia-ischemia, inflammation, and excitotoxicity in various species (mice, rats, pigs), magnesium sulfate preconditioning decreased the induced lesions’ sizes and inflammatory cytokine levels, prevented cell death, and improved long-term behavior. In humans, some observational studies have demonstrated reduced risks of cerebral palsy after antenatal magnesium sulfate therapy. Meta-analyses of five randomized controlled trials using magnesium sulfate as a neuroprotectant showed amelioration of cerebral palsy at 2 years. A meta-analysis of individual participant data from these trials showed an equally strong decrease in cerebral palsy and the combined risk of fetal/infant death and cerebral palsy at 2 years. The benefit remained similar regardless of gestational age, cause of prematurity, and total dose received. These data support the use of a minimal dose (e.g., 4 g loading dose ± 1 g/h maintenance dose over 12 h) to avoid potential deleterious effects. Antenatal magnesium sulfate is now recommended by the World Health Organization and many pediatric and obstetrical societies, and it is requisite to maximize its administration among women at risk of preterm delivery before 32 WG.

## Introduction

Preterm brain injury remains a crucial and unresolved issue among neonatologists. The ensuing cerebral lesions (i.e., brain injury related to encephalopathy of prematurity, including white matter injury, periventricular leukomalacia, and intraventricular/intraparenchymal hemorrhage) are strongly associated with later cerebral palsy and neurobehavioral developmental disorders. The mechanisms leading to these forms of brain injury are numerous and may include inflammation or ischemic insult. Numerous risk factors may be present before, during, and after birth (e.g., intra- and extra-uterine growth restriction, systemic inflammation, or perinatal hypoxia-ischemia). Although no single neuroprotective intervention is known to prevent preterm brain injury, neuroprotective strategies should be adopted to reduce the risk of neurodevelopmental anomalies in premature newborns. One such intervention is antenatal administration of magnesium sulfate (MgSO_4_) in women at risk of preterm birth. This mini review discusses the benefits of antenatal MgSO_4_ administration for fetal neuroprotection.

## Why is MgSO_4_ a Good Candidate for Neuroprotection?

### Biological Properties

Magnesium is the fourth most prevalent ion in the body and contributes to several physiological processes including storage, metabolism, and energy utilization. In the brain, magnesium is predominantly bound to chelators such as adenosine triphosphate (ATP) and is a cofactor in more than 300 enzymatic reactions (1, 2). Magnesium ions are essential for DNA, RNA, and protein synthesis. It contributes to glycolysis and ATP production and functions as a cell membrane stabilizer. In the central nervous system, magnesium is a non-competitive blocker of the *N*-methyl-d-aspartate (NMDA) glutamate receptor and modulates calcium influx. Its physiological role as a calcium channel blocker (3) and modulator of sodium and potassium flux through its action on ion pumps (e.g., Na+/K+ ATPase) and other membrane receptors (e.g., nicotinic acetylcholine receptor) (4) underlies its central role in heart function, muscle contraction, vascular tone, and nerve impulse conduction.

Sixty percent of magnesium is stored in bone, 20% in muscle, and 20% in soft tissue. Magnesium exists primarily in an ionized state (60%) but may also be complexed to proteins (33%) or anions (7%). Normal adult plasma concentration of magnesium is 0.75 mmol/L (95% confidence interval [CI]: 0.45–1.05) (5). In newborns, magnesium levels increase during the first week after birth (0.91 mmol/L [95% CI: 0.55–1.26]) (6).

### Potential Mechanisms of Action Underlying the Neuroprotective Effect of Magnesium

Multiple mechanisms may underlie the neuroprotective impact of magnesium. Magnesium affects several pathways potentially involved in preterm brain injury. As a non-competitive NMDA receptor antagonist, magnesium prevents excitotoxic calcium-induced injury (7). Magnesium decreases extracellular glutamate under ischemic conditions, possibly reducing excitotoxicity (8). Magnesium limits calcium influx through voltage-gated channels, which may reduce the activation of apoptosis (9).

Magnesium also has anti-inflammatory properties as it reduces oxidative stress and reduces the production of pro-inflammatory cytokines interleukin-6 and tumor necrosis factor-α (10–14). Magnesium deficiency increases endothelial nitric oxide production, which can promote endothelial dysfunction (15, 16). This could involve decreased calcium influx and activation of phagocytic cells, inhibition of neurotransmitter release, or inhibition of nuclear factor kappa B.

### Neuroprotective Effects of Magnesium in Preclinical Studies

Since the 1980s, animal studies have investigated the neuroprotective role of magnesium. Early experiments involved adult animal models of hypoxia, stroke, or traumatic brain injury. In 1984, Vacanti and Ames demonstrated neuroprotective effects of MgSO_4_ in an adult rabbit spinal cord ischemia model (17). In 1987, MgSO_4_ administration to rat hippocampal slices reduced the effect of hypoxia (18). McIntosh et al. demonstrated in 1989 that post-traumatic MgSO_4_ injection decreased neurological disorders in a dose-dependent manner (19). In 1996, Marinov et al. showed that MgSO_4_ administration before a focal ischemic episode in rats could be neuroprotective by blocking NMDA receptors (20). The neurological impact of MgSO_4_ on the developing brain was evaluated in several lesion models. In 1990, McDonald et al. showed that cerebral lesions induced by intraspinal injection of NMDA in postnatal (P) day 7 rats were decreased after intraperitoneal administration of MgSO_4_ (21). Several studies have reported the importance of the timing of MgSO_4_ administration. Intraperitoneal administration of MgSO_4_ reduced the excitotoxic brain lesions in mice induced by intracerebral injection of ibotenate (a glutamate receptor agonist) on P5. However, there was no effect on brain lesions developing on the day of birth or on P10 (brain lesions induced by intracerebral injection of ibotenate in mice are comparable to those identified in preterm human infants by age, specifically P0–22 weeks of gestation (WG), P2–26 WG, P5–32 WG, P10–41 WG) (22). In this P5 model, MgSO_4_ prevented sensorimotor alterations in P6 and P7 and prevented motor impairment, fine motor skill alteration, and memory deficits in adolescent mice (P34–40) (23). In the seminal model of focal hypoxia-ischemia established by the Rice-Vannucci procedure (surgical ligature of the right carotid artery followed by a 1–2-h exposure to 8% oxygen) in rats, MgSO_4_ injection before the hypoxic episode on P7 led to reduced lesion sizes, decreased hippocampal apoptosis, and improved adult sensorimotor performances (9, 24). In that model, MgSO_4_ treatment preserved mitochondrial respiration and reduced inflammation, thus reducing the production of reactive oxygen species after hypoxia-ischemia (16).

Under hypoxic conditions (fraction of inspired oxygen 5–7%) in P2 piglet brains, MgSO_4_ prevented the changes induced by hypoxia in the function of neuronal nuclear membrane, which decreased the transcription of apoptotic proteins and kinase activity. These actions ultimately prevented programmed cell death (25, 26). The neuroprotective effect of MgSO_4_ was also assessed under inflammatory conditions. In pregnant rats, lipopolysaccharide (LPS)-induced inflammation affected progeny learning and memory capabilities at 3 months, which is improved by antenatal MgSO_4_ treatment (27). MRI abnormalities (increased T2 and diffusion coefficient levels in white and gray matter) were highlighted for pups of LPS-treated dams, consistent with diffuse cerebral injury, which may be prevented by antenatal MgSO_4_ treatment (28). MgSO_4_ protected oligodendrocyte lineage cells *in vitro* in a model of hypoxic-ischemic injury (29).

## Effects of MgSO_4_ Treatment in Pregnancy

### Use of MgSO_4_ in Obstetrics for Maternal Indication

MgSO_4_ has been used in obstetrics for decades as a tocolytic agent and for prevention or treatment of seizures in women with preeclampsia or eclampsia (30, 31). Despite strong evidence indicating effectiveness in preventing eclampsia, MgSO_4_ is ineffective in delaying preterm birth (32). Despite weak evidence, MgSO_4_ is still recommended by the American College of Obstetricians and Gynecologists for short-term pregnancy prolongation (up to 48 h) to allow the administration of corticosteroids (33). In a European population-based cohort study, 35% of women with severe pre-eclampsia, eclampsia, or HELLP syndrome received MgSO_4_ before delivery. Only 1 of 119 hospital units reported using MgSO_4_ as a first-line tocolytic (34).

### MgSO_4_ Transplacental Passage

Fetuses are passively exposed to MgSO_4_ administered to pregnant women. In animals, fetal blood magnesium concentrations increase after maternal administration (35–37) and correlate with maternal blood levels (38, 39). The ratio of the mean fetal magnesium level to the mean maternal serum level at delivery was estimated at 0.94 ± 0.15 (40).

## Observational Studies

Considering its use in obstetrics for maternal indications, its transplacental passage, and its neuroprotective action in animal studies, several observational studies have focused on the impact of MgSO_4_ on neurological outcomes in preterm neonates. Nelson and Grether showed that exposure to MgSO_4_ exposure was higher in the control group than in the group of children with cerebral palsy (odds ratio [OR], 0.14; 95% CI, 0.05–0.51) (41). In another cohort study, prenatal MgSO_4_ exposure was associated with a reduced risk of cerebral palsy at 3–5 years (OR, 0.11; 95% CI, 0.02–0.81) (42). Other observational studies have not shown effects of MgSO_4_ on infant neurological outcomes (43–52). A meta-analysis of these observational studies highlighted that antenatal MgSO_4_ treatment was associated with a significantly reduced risk of mortality (risk ratio [RR], 0.73; 95% CI 0.61–0.89) and cerebral palsy (OR, 0.64; 95% CI 0.47–0.89) (53). Antenatal MgSO_4_ treatment was also associated with a decreased incidence of apparent echodensities and echolucencies on neonatal cranial ultrasonography and cerebellar hemorrhage on MRI (54, 55).

## Randomized Controlled Trials (RCTs) of Magnesium as a Neuroprotectant

A total of five RCTs were performed in the 1990s and 2000s. Notably, two RCTs are ongoing: MASP (for administration of antenatal magnesium sulfate for the prevention of cerebral palsy in preterm infants, inclusion at 24–32 WG) and MAGENTA (inclusion at 30–34 WG) (56, 57).

### Magnesium and Neurological Endpoints Trial (MagNET)

A total of 1,049 women in preterm labor at 25–33 WG (165 fetuses) treated at a single US center between October 1995 and January 1997 were included in the MagNET. Cases of triplet pregnancy or chorioamnionitis were excluded. In the tocolytic arm, women in active labor with cervical dilatation of at least 4 cm were randomly allocated to receive MgSO_4_ (4 g bolus then 2–3 g/h maintenance dose) or another tocolytic agent. In the neuroprotection arm, women with cervical dilatation of more than 4 cm were randomly allocated to receive MgSO_4_ (4 g bolus only) or 0.9% saline placebo.

The study was stopped prematurely in January 1997 due to significant mortality in the MgSO_4_ group (58, 59) and was widely discussed (60–63). The excessive number of mortalities occurred primarily in the tocolytic arm. The mortality rate in the MgSO_4_ group (11%) was consistent with that in previous reports of premature infants, whereas that in the placebo group was unusually low (1.4%). Moreover, causes of death were similar to those typical among premature children and were therefore difficult to attribute solely to MgSO_4_ treatment. Additionally, the confounding impact of multiple births was not accounted for, as more twin neonates were assigned to the treatment group than the placebo group. Finally, this increased mortality rate conflicted with the results of observational studies.

### Australasian Collaborative Trial of Magnesium Sulfate (ACTOMgSO_4_)

A total of 1,062 women in preterm labor before 30 WG from 16 centers were included in the Australasian Collaborative Trial of Magnesium Sulfate (ACTOMgSO_4_) between February 1996 and September 2000 (64). MgSO_4_ (4 g bolus followed by 1 g/h maintenance for 24 h or until birth) was randomly allocated to 535 women (629 live fetuses), and 527 women (626 live fetuses) received placebo. Although the primary study outcome, the rate of cerebral palsy at 2 years, was similar between the groups (5.7% in the MgSO_4_ group versus 6.7% in the control group; RR, 0.85; 95% CI, 0.55–1.31), the rate of motor dysfunction was significantly lower in the MgSO_4_ group (2.9 versus 5.4% in the control group; RR, 0.53; 95% CI, 0.30–0.92). Neonatal and pediatric mortality rates were also similar.

### PREMAG Trial

The PREMAG trial included 573 women treated at 18 French centers between July 1997 and July 2003 (65), with 286 women (354 fetuses) randomly assigned to receive a 4-g bolus of MgSO_4_ and 278 women (341 fetuses), placebo. The trial was stopped after 6 years of enrollment. The primary outcomes (the rates of white matter injury and mortality) were similar between the groups (white matter injury, 10% versus 11.7%; OR, 0.78; 95% CI, 0.47–1.31; mortality, 9.4 versus 10.4%; OR, 0.79; 95% CI, 0.44–1.44). Combined death or gross motor dysfunction at 2 years was lower in the MgSO_4_ group (25.6 versus 30.8%; OR, 0.62; 95% CI, 0.41–0.93), but there was no difference in cerebral palsy (66).

### Beneficial Effects of Antenatal Magnesium Sulfate (BEAM)

The BEAM trial included 2241 women in preterm labor before 32 WG at 20 centers between December 1997 and May 2004 (67). Women were randomized to receive a 6-g bolus of MgSO_4_ followed by a 2-g/h maintenance dose for 12 h (1,096 women, 1,188 fetuses) or placebo (1,145 women, 1,256 fetuses). Antenatal MgSO_4_ administration had no impact on pediatric mortality. Although the primary outcome (composite of stillbirth or death by 1 year or cerebral palsy at 2 years) was similar in the two groups, moderate or severe cerebral palsy was significantly reduced in the MgSO_4_ group (1.9 versus 3.5%; RR, 0.55; 95% CI, 032–0.95).

### MAGnesium Sulfate for Prevention of Eclampsia (MAGPIE)

The MAGPIE trial, a large international trial to evaluate the impact of antenatal MgSO_4_ administration in the prevention of eclampsia, included 10,141 women with preeclampsia between July 1998 and November 2001: 1,544 women (1,593 fetuses) before 37 WG (68). The women were randomly allocated to receive either MgSO_4_ (4 g bolus followed by 1 g/h maintenance dose for 24 h) or placebo. A pediatric follow-up study including 4,483 children (2,254 and 2,229 in the MgSO_4_ and placebo groups, respectively) showed no difference in neurological outcomes (Ages and Stages questionnaire) or mortality at 18 months. Notably, only 19% of the children followed were born before 33 WG.

### Meta-Analyses

These five RCTs have been the subject of four meta-analyses to date, with consistent findings and conclusions (69–73). In all meta-analyses, antenatal MgSO_4_ given to women at risk of preterm delivery was associated with a significantly reduced risk of cerebral palsy in children exposed *in utero*, with an RR ranging from 0.61 to 0.70 and no impact on mortality. The number of women needed to treat (NNT) to prevent one case of cerebral palsy ranged from 56 to 74 in infants born before 34 WG, and it was 29 in those born before 28 WG (Table 1). Minor maternal side effects (e.g., flushing, nausea or vomiting, sweating, injection site discomfort) were more frequent in the MgSO_4_ groups, but with no significant effect on serious maternal complications.

**Table 1:** Main outcomes of the meta-analyses.

	Pediatric mortality^a^	Cerebral palsy^a^	Death or cerebral palsy^a^	Number needed to treat to avoid 1 CP^b^
Doyle et al. (70)	1.04 (0.92–1.17)	0.68 (0.54–0.87)	0.94 (0.78–1.12)	63 (43–155)
Conde-Agudelo and Romero (71)	1.01 (0.89–1.14)	0.69 (0.55–0.88)	1.01 (0.89–1.14)	74 (41–373)
Costantine et al. (72)	1.01 (0.89–1.14)	0.7 (0.55–0.89)	0.92 (0.83–1.03)	Before 30 WG: 46 (26–187) Between 32 and 34 WG: 56 (34–164)
Zeng et al. (73)	0.92 (0.77–1.11)	0.61 (0.42–0.89) (moderate to severe CP)	N/A	N/A
Crowther et al. (IPD meta-analysis) (74)	1.03 (0.91–1.17)	0.68 (0.54–0.87)	0.86 (0.75–0.99)	46 (CI not shown)

*^a^Relative risk (95% CI)*.

*^b^Number needed to treat (95% CI)*.

*CP, cerebral palsy; CI, confidence interval; IPD, individual participant data*.

An individual participant data meta-analysis was also undertaken by the AMICABLE group (Antenatal Magnesium sulfate Individual participant data international Collaboration: Assessing the benefits for babies using the Best Level of Evidence) to explore the interaction between treatment and participant characteristics (74), which included the 5 above-mentioned RCTs (5,493 women and 6,131 babies). The overall RR of cerebral palsy among survivors after antenatal MgSO_4_ was 0.68 (95% CI, 0.54–0.87), and the NNT was 46. Interestingly, MgSO_4_ also reduced the combined risk of fetal/infant death or cerebral palsy in the analysis of the 4 trials with fetal neuroprotective intent (RR 0.86, 95% CI, 0.75–0.99).

In all RCTs and meta-analyses to date, MgSO_4_ treatment had no impact on pediatric mortality or neonatal morbidity (respiratory distress syndrome, chronic lung disease, any intraventricular hemorrhage, cystic periventricular leukomalacia, necrotizing enterocolitis, patent ductus arteriosus, and retinopathy of prematurity). Similarly, MgSO_4_ treatment was not associated with serious maternal side effects. The benefit remained constant regardless of gestational age, cause of prematurity, total dose received, or maintenance dose administration after the loading dose. These data indicating persistent benefits of MgSO_4_ regardless of dose and support the use of low doses (e.g., 4 g loading dose ± 1 g/h maintenance dose for 12 h, 16 g maximum total dose) compared to high doses (e.g., 6 g loading dose + 2 g/h maintenance dose during 24 h, maximum total dose received: 54 g). Indeed, high MgSO_4_ dosage was implicated in the vasculopathy and high mortality observed in the MagNET trial (75). In a mouse preclinical model, MgSO_4_ demonstrated a dose-dependent, potentially deleterious effect on brain angiogenesis, vessel damage, and endothelial cell survival. The highest neuroprotective dose of MgSO_4_ induced cerebral hypoperfusion, whereas the lowest dose did not (76). These results support the use of MgSO_4_ in low doses.

### Long-Term Follow-Up

Cohorts from the PREMAG and ACTOMgSO_4_ trials were followed up over their school-age years. From the PREMAG trial, 431 children were assessed at a mean age of 11 years (26.9% lost to follow-up) using a questionnaire completed by the parents (77). Although the ORs for motor, cognitive, behavioral outcomes, and school performance were favorable after magnesium treatment, the impact on neurodevelopment was not statistically significant. From the ACTOMgSO_4_, 669 children (21.3% lost to follow-up) were assessed at a mean age of 8 years using pediatric and psychological assessments and questionnaires completed by parents and teachers (78). Antenatal MgSO_4_ treatment had no impact on neurological, cognitive, behavioral, or school-related outcomes. Neither the PREMAG study nor the ACTOMgSO_4_ showed any effect on cerebral palsy at 2 years, likely because of limited sample sizes. Only the larger BEAM trial and meta-analyses reported reductions in cerebral palsy at 2 years. These long-term follow-up studies detected no harmful effects after antenatal MgSO_4_ treatment, although they were not designed for this purpose.

## Implementation of Magnesium in Neuroprotective Protocols Worldwide

In France, in 2015, only 60% of tertiary maternity hospitals used MgSO_4_ for fetal neuroprotection, with protocols that differed by maximum gestational age, possibility of retreatment, and monitoring (79). In Europe, in 2012, only 9 of 119 tertiary maternity hospitals (7.6%) used MgSO_4_ for fetal neuroprotection (34). Lack of experience and an absence of a written protocol or national guidelines, decision-making processes, environmental contexts, or beliefs about possible consequences seemed to represent barriers to widespread applications of MgSO_4_ in women at risk of preterm delivery (79, 80). Studies assessing MgSO_4_ protocol implementation found that nearly 70% of eligible women received MgSO_4_ before preterm delivery, and approximately 90% delivered within 24 h. The main reasons for not giving treatment were omission by the medical team and urgent delivery (81, 82). In an Australasian audit, the proportion of eligible women not receiving MgSO_4_ decreased significantly after publication of national guidelines, from 69.7% in 2010 to 26.9% in 2011, which was maintained in 2012 and 2013 (22.5%) (83). In Canada, a knowledge translation strategy (including national practice guidelines, online e-learning modules, educational rounds, and evaluation of barriers and feasibility) was associated with an 84% increase in optimal MgSO_4_ use (84). To improve the rates of MgSO_4_ administration to eligible women, implementing educational programs could be effective.

## Conclusion

Preterm birth is a major cause of death and a significant cause of long-term disability worldwide (85). MgSO_4_ is a safe and effective molecule that plays a key role in protecting the immature brain. It is a cost-effective, feasible, efficient, and safe intervention that contributes to the improvement of neurological outcomes. While MgSO_4_ has not been found to significantly improve cognition and behavior outcomes at school age, it prevents cerebral palsy at 2 years. Its use is now recommended by several pediatric and obstetrical societies, as well as the World Health Organization (strong recommendation based on moderate-quality evidence) for women at risk of imminent preterm birth before 32 WG. More work is needed to clarify the impact of MgSO_4_ on the cognitive outcome and efforts to improve the MgSO_4_ coverage of eligible women should be reinforced.

## Author Contributions

CC, LS, and SM contributed equally to the writing of this review.

## Conflict of Interest Statement

The authors declare that the research was conducted in the absence of any commercial or financial relationships that could be construed as a potential conflict of interest.
